# E6-Encoded by Cancer-Causing Human Papillomavirus Interacts with Aurora Kinase A To Promote HPV-Mediated Carcinogenesis

**DOI:** 10.1128/jvi.01872-22

**Published:** 2023-01-30

**Authors:** Sile Li, Man Kin Yim, Ka Lai Yip, Chuanyun Xiao, Ho Yin Luk, Sijia Xiao, Zigui Chen, Paul K. S. Chan, Siaw Shi Boon

**Affiliations:** a Department of Microbiology, Faculty of Medicine, The Chinese University of Hong Kong, Hong Kong SAR, China; University of Toronto

**Keywords:** HPV, E6, Aurora kinase A, carcinogenesis, cell cycle checkpoints

## Abstract

The expression of human papillomavirus (HPV) oncoproteins perturbed multiple cellular events of the host cells, leading to the formation of cancer phenotypes. Our current and previous studies indicated that Aurora kinase A (AurA), a mitotic regulator that is often aberrantly expressed in human cancers, is preferentially bound to E6-encoded by cancer-causing HPV. AurA is believed to be important for the proliferation and survival of HPV-positive cells. Nonetheless, the interaction between AurA and E6, and the mechanism of how this association is involved in carcinogenesis, have not been elucidated clearly. Hence, we performed a series of biochemical assays to characterize the AurA-E6 association and complex formation. We found the C-terminus of E6, upstream of the PDZ binding motif of E6, is important to forming the AurA-E6 complex in the nucleus. We also showed that the expression level of E6 corresponded positively with AurA expression. Meanwhile, the functional consequences of the AurA-E6 association to AurA kinase function and host cellular events were also delineated. Intriguingly, we revealed that AurA-E6 association regulated the expression of cyclin E and phosphor-Histone H3, which are involved in G1/S and mitotic phases of the cell cycle, respectively. Depletion of AurA also reduced the invasive ability of HPV-positive cells. AurA inhibition may not be sufficient to reduce the oncogenic potential exerted by E6. Altogether, our study unleashed the mechanism of how HPVE6 deploy AurA to promote cancer phenotypes, particularly through dysregulation of cell cycle checkpoints and suggests that the AurA-E6 complex possesses a therapeutic value.

**IMPORTANCE** We unveiled the mechanism of how HPV employs Aurora kinase A (AurA) of host cells to exert its oncogenic capability synergistically. We systematically characterized the mode of interaction between E6-encoded by cancer-causing HPV and AurA. Then, we delineated the consequences of AurA-E6 complex formation on AurA kinase function and changes to cellular events at molecular levels. Using a cell-based approach, we unleashed that disruption of AurA-E6 association can halt cancer phenotype exhibited by HPV-positive cancer cells. Our findings are vital for the designing of state-of-the-art therapies for HPV-associated cancers.

## INTRODUCTION

The ability of human papillomavirus (HPV) in causing cancers, including cancers that arise from the uterine cervix, oropharyngeal, vulvar, penile, and other anogenital regions, is through the combined action of the viral E6 and E7 oncoproteins. The multifunctional E6 and E7 proteins can degrade tumor suppressors of host cells, namely, p53 (1), Bak and PDZ containing proteins (2) by E6, and retinoblastoma (pRB) by E7 (3). In addition, both of the viral oncoproteins perturbed cellular events, including upregulation of AKT and mammalian target of rapamycin (mTOR) signaling pathway (4–6), as well as cell cycle (7, 8), leading to cell growth in an anchorage-independent manner (9), enhanced cell proliferation, and survival (6, 10, 11). The so-called “high-risk”- (hr-) HPVs can cause malignancy, while the “low-risk”- (lr-) HPV types cause benign warts and lesions. One of the prominent differences between E6-encoded by hr- and lr-HPV is the PDZ binding motif (PBM) located at the extreme C-terminal of hr-HPVE6s, whereas this motif is lacking in lr-HPVE6s.

Activation of AKT and mTOR cascade leads to activation of its downstream Aurora kinases, which comprises of Aurora kinase A (AurA), B (AurB), and C (AurC). AurA is involved in mitotic entry, centrosome maturation, separation, spindle assembly, and spindle damage repair (12). Upregulation of Aurora kinases is often implicated in human cancers, including cancers that arise from the uterine cervix and head and neck region (13), allowing cells to gain epithelial-to-mesenchymal transition (EMT), aberrant mitosis, and enhanced cell migration (14, 15). The previous study revealed that HPVE6 interacts with AurA (16), and AurA is required for the survival and proliferation of HPV-positive cells (17, 18). However, it remains unknown whether AurA is involved in further promoting HPV-mediated carcinogenesis.

Even though the association of AurA and E6 have been studied, the previous studies did not delineate whether the interaction between AurA and E6 is direct. In addition, it is also unclear whether AurA is involved directly in promoting HPV-mediated carcinogenesis. In this study, we utilized a series of biochemical assays to assess the amino acid of E6 important for the AurA-E6 complex formation. We also studied whether AurA interaction with E6 is conserved among the hr-HPVs, or lr-HPVE6 also can interact with AurA. Then, we delineated whether the E6 deploys AurA in HPV-containing cells to promote carcinogenesis.

## RESULTS

### HPV16E6 interacts with AurA at a stronger affinity than HPVE7.

**FIG 1:**
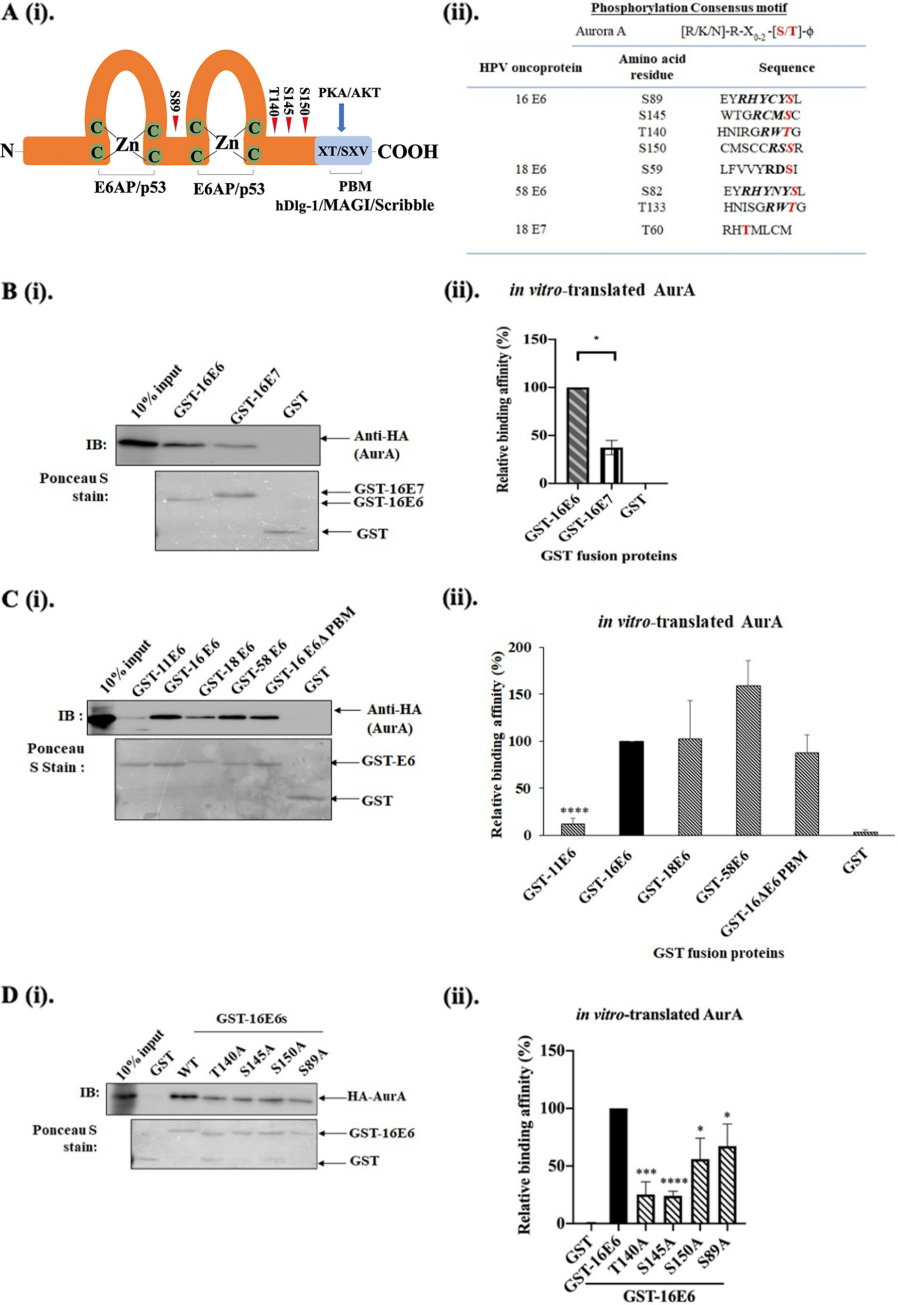
Preferential direct binding of Aurora kinase A (AurA) to the C-terminus of E6-encoded by HPV16. (A) (i). Schematic diagram shows the basic structure of cancer-causing HPV16E6 and potential amino acids recognized by Aurora kinase A (AurA). E6 contains two major functional domains: two zinc (Zn) finger binding domains, the regions where E6 forms complex with E6AP and p53; and the unique dual-functional PDZ binding motif (PBM) at its extreme C-terminal end, the region where E6 binds to PDZ proteins [Disc large protein-1 (Dlg-1), membrane-associated guanylated kinase inverted (MAGI) protein and Scribble)], and which is also a target for PKA/AKT phosphorylation. (ii). The right panel shows the AurA recognition motif [R/K/N]-R-X_0-2_-X-[S/T]-ϕ. Also shown are the key amino acids within E6-encoded by HPV16, 18 and 58, and E7-encoded by HPV18 predicted using Scansite that can be recognized by AurA: HPV16E6 at residues S89, T140, S145 and S150 of HPV16E6; HPV18E6 at residue S59; HPV58 at residues S82 and T133; HPV18E7 at residue T60. (B) (i). An *in vitro* binding assay was performed by incubating the indicated purified GST fusion proteins with *in vitro*-translated Aurora kinase A (AurA). After extensive washing, the bound AurA protein was detected via Western blotting using an HA-specific antibody. The immunoblot (IB) on the upper panel shows the interaction of AurA with GST-16 E6 and E7, while the lower panel shows the Ponceau S stained of the blot. (ii). The bar graph shows the quantitation of the relative level of AurA bound to GST fusion proteins indicated from 3 independent experiments (*n* = 3). Quantitation was performed using ImageJ software and statistical analysis was performed using Prism. C (i). A similar *in vitro* binding assay was performed, in which *in vitro*-translated HA-AurA protein was incubated with purified GST-tagged HPV-11E6 (GST-11E6), -16E6 (GST-16E6), -18E6 (GST-18E6), -58E6 (GST-58E6) and 16E6ΔPBM (GST-16 E6ΔPBM) as indicated. The immunoblot (IB) on the upper panel shows the interaction of AurA with GST-tagged fusion proteins, while the lower panel shows the Ponceau S stained of the blot. (ii). The bar graph shows the binding affinity of AurA to GST-E6 fusion proteins relative to GST-16E6 wild type. The intensities of the bands were analyzed using ImageJ (*n* = 3). All data are presented as means ± standard error of the mean (SEM). (*, *P* < 0.05, ****, *P* < 0.0001). D (i). An *in vitro* binding assay was performed, in which *in vitro*-translated HA-AurA protein was incubated with purified GST-tagged HPV16E6 wild types (GST-16E6) and mutants (T140A, S145A, S150A, and S89A), as indicated. After extensive washing, the protein complexes were subjected to Western blotting. The upper panel showed the immunoblot (IB) incubated with HA-specific antibody to detect the expression level of AurA, while the lower panel showed the Ponceau S stain of the blot. (ii). The bar graph shows the binding affinity of AurA to GST-E6 mutants relative to GST-16E6 wild type. The intensities of the bands were analyzed using ImageJ (*n* = 3). All data are presented as means ± standard error of the mean (SEM). (*, *P* < 0.05; ***, *P* < 0.001; ****, *P* < 0.0001).

We predicted using the Scansite freeware (19) that E6 and E7 oncoproteins encoded by HPV16, 1,8 and 58 harbors AurA recognition motif, as shown in Fig. 1A. We verified if AurA is a genuine interacting partner of the HPV oncoproteins. To address this, we performed *in vitro* binding assay, in which *in vitro*-translated AurA was incubated with purified GST-tagged E6 or E7 fusion proteins. As HPV16 contributes to the vast majority of human cancers, in this part, we focused on comparing the binding affinity between E6- and E7-encoded by HPV16 to AurA. As shown in Fig. 1B, GST-tagged E7-encoded by HPV16 (37.33% ± 7.4%, *P* < 0.05) bound to AurA at a weaker affinity than GST-tagged E6-encoded by HPV16, indicating the preferential direct binding of AurA to E6- than E7-encoded by HPV16.

### AurA recognition is more conserved among E6 encoded by cancer-causing HPV types, and independent of E6-PBM.

We then studied if E6-encoded by both hr-HPV (HPV16, 18, and 58) and lr-HPV (HPV11) interact with AurA. We performed a similar *in vitro* binding assay. From the immunoblot (Fig. 1C), the *in vitro*-translated AurA bound to GST-tagged E6-encoded by 18 (102% ± 41.3%, *P* > 0.05) and 58 (158% ± 27.2%, *P* > 0.05) at a similar affinity compared with GST-tagged E6-encoded by HPV16, whereas lr-HPV11E6 (12% ± 5.6%, *P* < 0.0001) had a much lower binding affinity to AurA. In order to understand if the E6-PDZ binding motif (PBM) conserved among the hr-HPV is important for AurA-E6 complex formation, we also included purified GST-tagged HPV16E6 with its PBM deleted (E6ΔPBM) in the *in vitro* binding assay. We were intrigued to find that GST-16E6 ΔPBM (87% ± 19.0%, *P* > 0.05) bound to AurA at an affinity similar to that of GST-16E6 wild type (Fig. 1C). Taken together, these results indicated that AurA binds strongly to E6-encoded by hr-HPVs, but not lr-HPV. The interaction between AurA and E6 occurs in an E6-PBM-independent manner.

Next, we wanted to further investigate which amino acid residue within E6 is recognized by AurA. We generated E6 mutants with the four amino acids of HPV16E6 predicted to be recognized by AurA, namely, serine (S) 89, threonine (T) 140, and S145 and S150 to alanine (A). Then, we performed a similar *in vitro* binding assay, in which *in vitro*-translated AurA was incubated with purified GST-tagged E6 full length and mutants. Intriguingly, as shown in Fig. 1D, compared with the binding affinity of E6 wild type, we observed a decreased binding affinity of E6T140A (25.3% ± 6.4%, *P* < 0.001) and S145A (24.0% ± 2.3%, *P* < 0.0001) to AurA. Moreover, a decrease was observed in binding affinity of E6S89 (67.3% ± 11.05%, *P* < 0.05) and E6S150 (56.0% ± 10.4%, *P* < 0.05) to AurA relative to E6 wild type. Altogether, our results indicate that both HPV16E6 wild type and HPV16E6ΔPBM bind directly to AurA. Despite the residues S89 and 150 of E6 may be important for E6 binding AurA, residues T140 and S145 are more critical for AurA and HPV16E6 to form complex.

### AurA forms complex with E6 predominantly in the nucleus.

**FIG 2:**
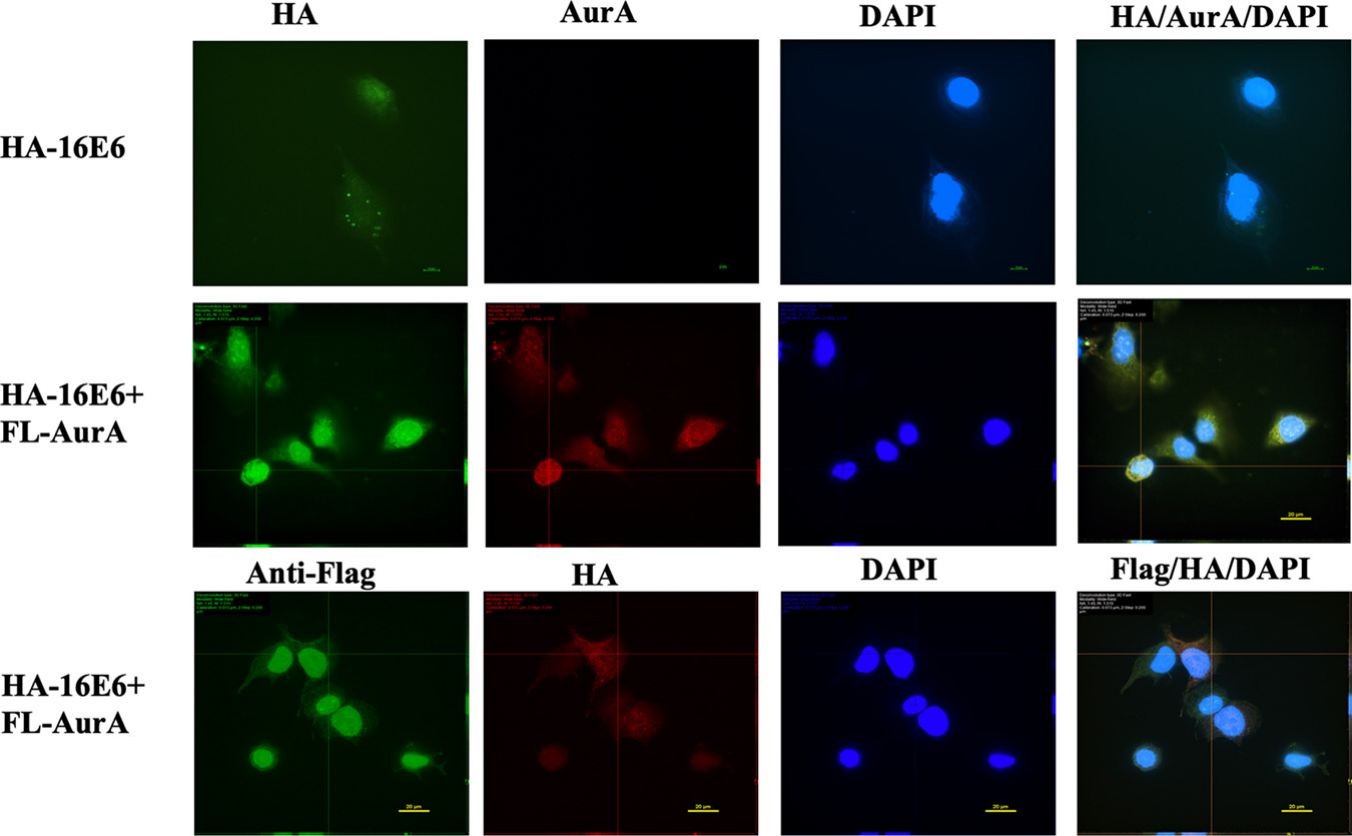
Aurora kinase A colocalizes with 16E6 predominantly in the nucleus. U-2 OS cells were transfected with pcDNA3.1: Flag-AurA (FL-AurA) and pGW1: HA-16E6 (HA-16E6) plasmids. The cells were fixed and incubated with primary antibodies (anti-AurA, anti-Flag and anti-HA), followed by the incubation with the relevant Alexa Fluor 568-conjugated anti-rabbit and Alexa Fluor 488-conjugated anti-mouse secondary antibodies. The cells were then counterstained with 4,6-diamidino-2-phenylindole (DAPI). The Z-staking images for subcellular expression of AurA (Red) and E6 (Green) were examined using the Nikon fluorescence microscope.

We next wanted to investigate the cellular compartment where AurA forms a complex with E6 in the cells. We transfected an HPV-null osteosarcoma cell line, U-2 OS with Flag-tagged AurA and HA-tagged E6. The cells were fixed and proceeded with immunofluorescence staining using HA, Flag, and AurA-specific antibodies. When E6 and AurA were co-expressed in U-2 OS cells, the co-staining was observed predominantly in the nucleus of the cells (Fig. 2), suggesting that AurA-E6 complex formed predominantly in the nucleus.

### The binding of E6 to AurA is important for AurA kinase activity.

**FIG 3:**
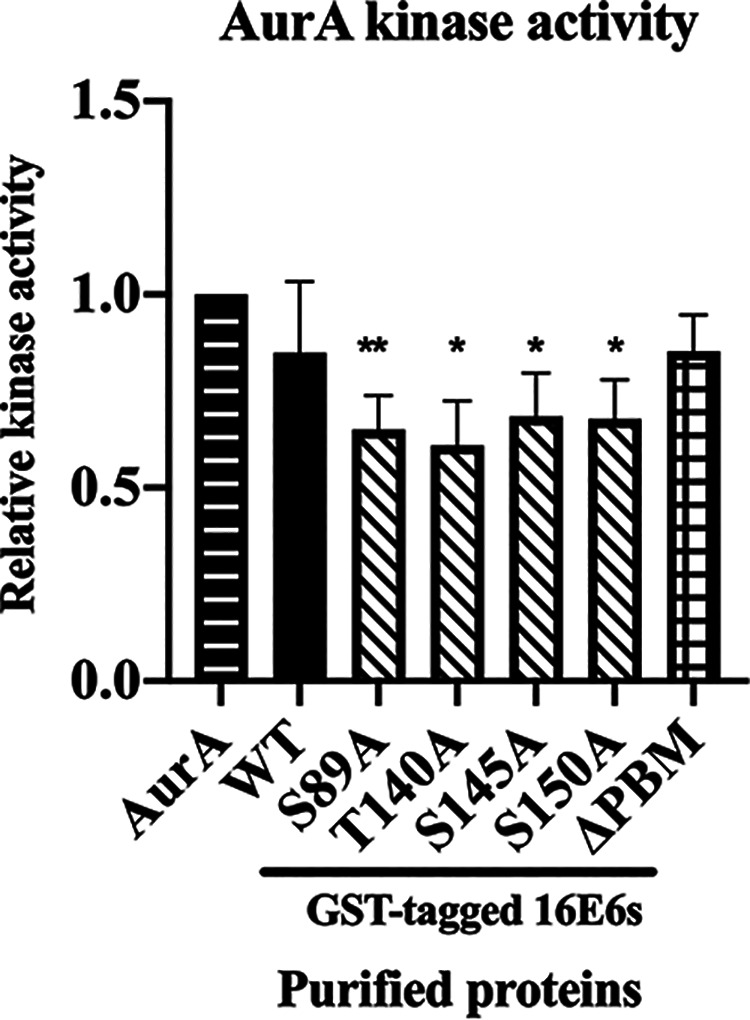
The interaction between Aurora kinase A and E6 may not affect the integrity of AurA and its kinase function. ADP-Glo assay was performed to determine the kinase activity of Aurora kinase A (AurA). The indicated purified GST-tagged 16E6 fusion proteins were incubated with purified AurA. The ability of AurA to convert ATP into luminescent ADP was measured. The bar graph shows the percentage (%) of AurA activity normalized with the amount of GST fusion proteins in the respective experiments. (*n* = 3). All data are presented as means ± standard error of the mean (SEM). (*, *P* < 0.05; **, *P* < 0.01). The data were evaluated by comparing the level of phosphorylation between AurA (autophosphorylation) with GST-tagged HPV16E6 wild types (WT) and mutants (T140A, S145A, S150A, S89A, and ΔPBM).

AurA is one of the major serine-threonine kinases involved in regulating the cell cycle. We wanted to delineate whether E6 binding to AurA could affect its kinase activity. We performed a sensitive luminescence-based ADP-Glo kinase assay. This assay examined the level of consumed ATP (ADP), with the higher the amount of ADP detected signifying the accelerated kinase activity of a kinase, and *vice versa*. We incubated purified AurA with purified GST-tagged E6 fusion proteins. As AurA can undergo autophosphorylation, purified AurA was incubated with purified GST protein alone was included and served as a positive control. As shown in Fig. 3, the presence of GST-E6 and E6 ΔPBM did not affect the AurA kinase activity, (0.85 ± 0.18, *P* = 0.445) and (0.85 ± 0.05, *P* = 0.063). While the AurA kinase activity was reduced in the presence of GST-E6 mutants, which demonstrated reduced binding affinity to AurA: S89A (0.65 ± 0.09, *P* < 0.01), T140A (0.61 ± 0.12, *P* < 0.05), S145A (0.68 ± 0.12, *P* < 0.05), and S150A (0.68 ± 0.12, *P* < 0.05). This result showed that binding of E6 to AurA may not affect the integrity of AurA and its kinase function. While the presence of E6 mutants that are defective in establishing an association with AurA disrupts the kinase function of AurA.

### AurA enhances the expression of E6 protein, and vice versa.

**FIG 4:**
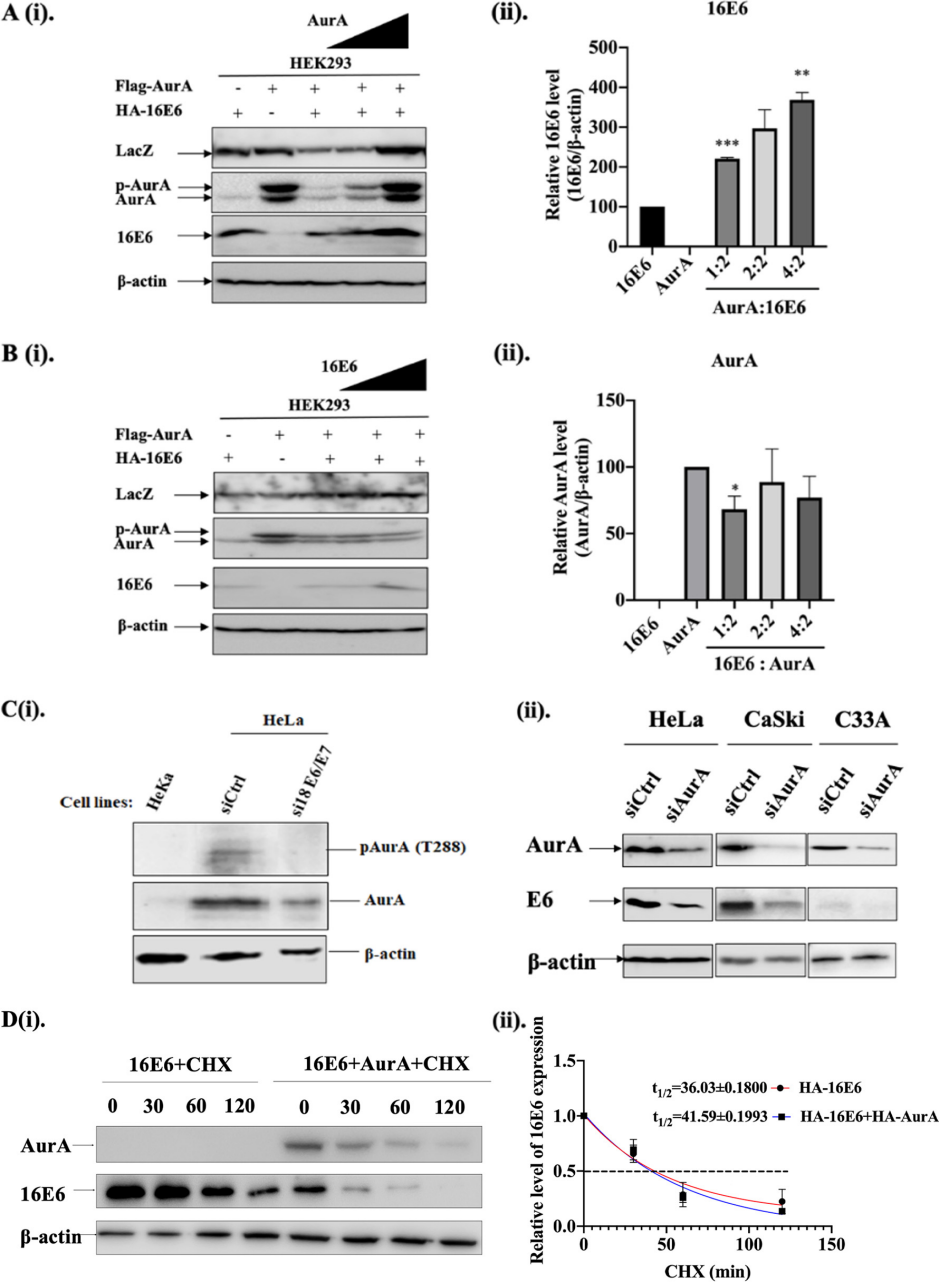
AurA upregulates the level of 16E6 protein and *vice versa*. (A) (i). HEK 293 cells were transfected with an increasing amount of pcDNA3.1: Flag-AurA (0.5 μg, 1 μg, and 2 μg) and amount of pGW1: HA-16E6 (1 μg). The cells were collected and total proteins were extracted. These proteins were then subject to Western blotting and the immunoblots were detected using β-galactosidase (LacZ) (a transfection efficiency control), AurA, anti-HA (for HPV16E6) and β-actin (a loading control) antibodies. (ii). The relative level of 16E6 co-expressed with increasing concentration of AurA was normalized with the expression level of 16E6 expressed alone and β-actin. The intensities of the bands were analyzed using ImageJ. Data are presented as means ± standard error of the mean (SEM) relative to 16E6. (**, *P* < 0.01; ***, *P* < 0.001). (B) (i). HEK293 cells were transfected with an increasing amount of pGW1: HA-16E6 (0.5 μg, 1 μg, and 2 μg) and fixed the amount of pcDNA3.1:Flag-AurA (1 μg). The cell were collected and the expression levels of LacZ, AurA, anti-HA and β-actin were ascertained using the respective specific antibodies. (ii). The relative level of AurA co-expressed with increasing concentration of 16E6 was normalized with the expression level of AurA expressed alone and β-actin. The intensities of the bands were analyzed using ImageJ. (*, *P* < 0.05). (C) (i). Total protein lysates were extracted from cells: HeKa (HPV-null primary keratinocytes which is a natural reservoir of HPV) and HeLa (HPV18 cervical cancer cells) transfected with siRNA against control (siCtrl) or 18 E6 and E7 (si18 E6/E7) for 72 h. AurA protein were ascertained by Western blotting using phospho-AurA [(pAurA) (T288)] and total AurA (AurA)-specific antibodies. Intriguingly, the levels of both pAurA and AurA decreased in HeLa cells with HPV18 E6/E7 downregulated (si18 E6/E7). β-actin was included as a loading control. (ii). Total protein lysates were extracted from cells: HeLa (HPV18-positive cervical cancer cells), CaSki (HPV16-positive cervical cancer cells) and C33A (HPV-null cervical cancer cells) transfected with siRNA against control (siCtrl) or AurA (siAurA) for 72 h. AurA protein were ascertained by Western blotting using anti-AurA, E6 and β-actin (a loading control) antibodies. Note the levels of E6 decreased in HeLa, CaSki and C33A cells with AurA downregulated (siAurA). (D) (i). HEK 293 cells were transfected with pGW1: HA-16E6 and/or pcDNA3.1:HA-AurA for 24 h and incubated with cycloheximide (CHX) at different time points as indicated. Total protein lysates were extracted from cells and subjected to Western blotting using the anti-HA (for HA-AurA and HA-16E6) and β-actin (a loading control) antibodies. (ii). The curve shows the protein level of 16E6 with or without the overexpression of AurA. The stability of 16E6 was analyzed by the one-phase exponential decay using GraphPad Prism.

Knowing that E6 can degrade its target proteins in a proteasome-dependent manner, like p53 and PDZ-containing proteins, we then asked if E6 expression could affect the expression of AurA proteins. We assessed the expression level of AurA when co-expressed with E6, and vice versa, via Western blotting. We transfected HEK293 cells with Flag-tagged AurA at an increasing amount, and a fixed amount of HA-16E6 at ratios of 1:1, 1:2, and 2:1; or HA-tagged E6 to a fixed amount of Flag-tagged AurA at a ratio of 1:1, 1:2, and 2:1. From the immunoblots (Fig. 4A), we found that the level of E6 protein increased significantly when AurA:E6 was expressed at a ratio of 1:2 (221.0 ± 3.0%, *P* < 0.001) and 2:1 (368.5% ± 18.5%, *P* < 0.01). When an increasing amount of E6 was co-expressed with a fixed amount of Flag-tagged AurA, the level of AurA protein decreased when E6:AurA was expressed at a ratio of 1:2 (68.3% ± 9.8%, *P* < 0.05). However, the level of AurA protein was not affected when E6 and AurA were expressed at an E6:AurA ratio of 1:1 and 2:1 (Fig. 4B).

To further elucidate the relationship between AurA and E6, we downregulated E6 in HeLa cells using specific short-interference RNA (siRNA) targeting E6 and E7. The total cell lysate was resolved via Western blotting. Cell lysate extracted from HeKa (HPV-null primary keratinocytes) was included as a control. When E6 and E7 were downregulated in HeLa cells, we observed decreased levels of phosphor and total AurA protein expression (Fig. 4C [i]). Simultaneously, we also downregulated AurA in HeLa, CaSki, and C33A cells using specific siRNA targeting AurA. We observed a decreased level of E6 protein in HeLa and CaSki cells upon AurA downregulation (Fig. 4C [ii]).

Next, we asked if AurA affects the stability of E6. To do this, we transfected HEK 293 cells with HA-16E6 and/or HA-AurA for 24 h. Following this, the cells were treated with cycloheximide (CHX) for a duration of 0, 30, 60, and 120 min. From the immunoblots (Fig. 4D [i]), we quantitated the half-life of E6 in the presence or absence of AurA (Fig. 4D [ii]). When co-expressed with AurA, the half-life of E6 was higher (41.59 ± 0.1993 min) than when E6 was expressed alone (36.03 ± 0.1800 min). Taken together, these results indicated that AurA is not a degradation target of E6. More importantly, in HPV-positive cells, the expression of AurA is important to maintain the stability and high level of E6 protein. Meanwhile, the downregulation of HPV oncoproteins also decreases the expression and activity of AurA in HPV-positive cells, and vice versa, indicating a positive correlation in the levels of AurA and E6 proteins.

### AurA and E6 association perturbs cell cycle regulation of HPV-positive cells.

As AurA possesses an oncogenic role in human cancers, we then asked if the association between E6 and AurA could perturb host cellular events. We delineated this using two approaches: (i) downregulation of AurA using siRNA against AurA (siAurA) in HPV-positive CaSki cells; and (ii) ectopically expressed AurA and/or HPV16E6 in HPV-null C33A cells. We then investigated the levels of proteins important for the cell cycle regulation (cyclin E for G1/S phase; cyclin B for G2/M phase; and phosphorylated [Serine 10]/acetylated [Lysine 9] Histone H3 [p-Ac-Histone H3] and total Histone H3 for mitotic phase), cellular signaling (phosphor and total AKT and ERK1/2), EMT (Vimentin), and cell migration (MMP2).

**FIG 5:**
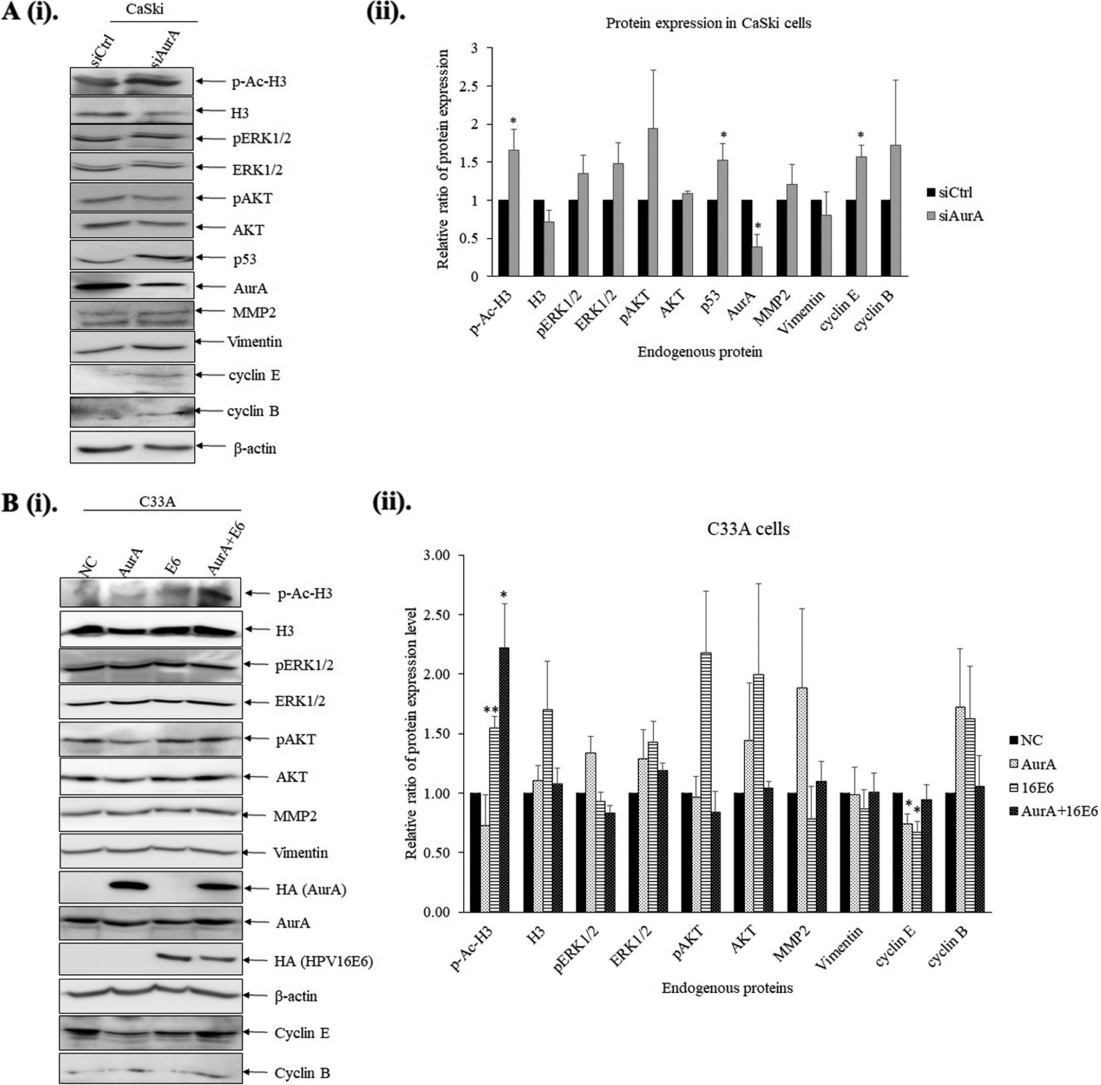
The association between AurA and E6 perturbs host cellular events. (A) (i). CaSki cells were transfected with short interference RNA (siRNA) against AurA or control (siCtrl) for 72 h. The protein extracts were subjected to Western blotting for the detection phosphorylated/acetylated-Histone H3 (p-Ac-H3), Histone H3 (H3), phosphor-ERK1/2 (pERK1/2), total ERK1/2, phosphor-AKT (pAKT), AKT, p53, AurA, metalloprotease protein 2 (MMP2), vimentin, cyclin E, cyclin B and beta (β)-actin specific antibodies. (ii). The relative expression levels of the cellular proteins relative to CaSki cells transfected with siCtrl were analyzed using ImageJ and GraphPad Prism (*n* = 3). (B) (i). The C33A cells were transfected mock-transfected (NC) or transfected with pcDNA3.1:HA-AurA (AurA) or/and pGW1: HA-16E6 (E6) plasmids. After 24 h, the total protein extracts were collected and subjected to Western blotting. The immunoblots were incubated with antibodies specific for phosphorylated/acetylated-Histone H3 (p-Ac-H3), Histone H3 (H3), phosphor-ERK1/2 (pERK1/2), total ERK1/2, phosphor-AKT (pAKT), AKT, HA (16E6), AurA, metalloprotease protein 2 (MMP2), vimentin, cyclin E, cyclin B and β-actin antibodies. (ii). The bar graph shows the expression levels of the proteins indicated relative to the mock-transfected group. The intensities of the bands were analyzed using ImageJ and GraphPad Prism (*n* = 3). All data were presented as means ± standard error of the mean (SEM). (*, *P* < 0.05; **, *P* < 0.01).

As shown in Fig. 5A, when the expression level of AurA was downregulated using specific siRNA against AurA (siAurA) in CaSki cells (0.38 ± 0.28, *P* < 0.05), we detected an increase in the level of phosphor-Histone H3 (1.65 ± 0.27, *P* < 0.05) and p53 (1.5 ± 0.37, *P* < 0.05) compared with CaSki cells transfected with siRNA against control (siCtrl) (Fig. 5A). The expression level of p53 was assessed as it serves as a surrogate marker of E6. The increased p53 protein expression indicates the downregulation of E6 in CaSki cells when AurA is depleted.

Meanwhile, when we ectopically expressed HA-tagged AurA and/or HA-tagged E6 in C33A cells, the level of phosphor-Histone H3 was increased when E6 was either expressed alone (1.55 ± 0.10, *P* < 0.01) or co-expressed with AurA (2.22 ± 0.37, *P* < 0.05) compared with mock-transfected cells (Fig. 5B). We also observed a reduced expression of cyclin E when AurA (0.74 ± 0.08, *P* < 0.05) or E6 (0.67 ± 0.09, *P* < 0.05) was expressed alone. The expression level of cyclin E in cells with AurA and E6 co-expressed was similar to the mock-transfected cells.

Using both approaches, either upon depletion of AurA or overexpression of AurA and E6, the expression levels of ERK1/2, AKT, MMP2, Vimentin, and cyclin B were increased when AurA and/or HPV16E6 were overexpressed, despite the increment was marginal. Altogether, these results consistently show that AurA and E6 association perturbs cell cycle checkpoints, mainly in regulating cyclin E and phosphor-Histone H3 during G1/S and mitotic phases, respectively.

### The differential efficacy of treatment of AurA inhibitors in HPV-positive cells.

**FIG 6:**
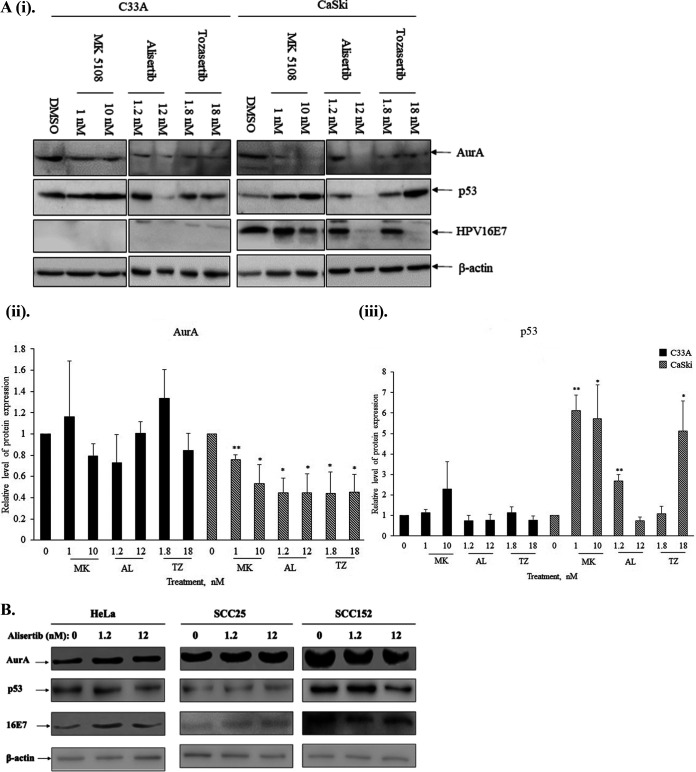
The treatment of Aurora kinase inhibitors in CaSki cells suppresses Aurora kinase A and HPV oncoproteins expression. (A) (i). C33A (HPV-null cervical cancer cells) and CaSki (HPV16-positive cervical cancer cells) were treated with potent Aurora kinase A inhibitor (MK5108 [ML] and Alisertib [AL]), or pan-Aurora kinase inhibitors (Tozasertib [TZ]) at concentrations indicated for 24 h. Simultaneously, the cells were also treated with DMSO, which served as vehicle control. The total cell lysate was collected and the expression levels of Aurora kinase A (AurA), p53 and 16E7 were ascertained via Western blotting. Beta(β)-actin was included as a loading control. The bar graphs show the mean expression levels of the (ii) AurA and (iii) p53 proteins indicated relative to the DMSO-treated sample. The intensities of the bands were quantitated using ImageJ software and GraphPad Prism. Error bars represent the mean ± standard error of the mean (SEM) (*n* = 3). (*, *P* < 0.05, **, *P* < 0.01). (B) HeLa (HPV18-positive cervical cancer cells), SCC25 (HPV-null human head and neck head and neck squamous cell carcinoma [HNSCC] cell line) and SCC152 (HPV-positive human HNSCC cell line) were treated with potent Aurora kinase A inhibitor (Alisertib) at concentrations indicated for 24 h. Simultaneously, the cells were treated with DMSO, which served as vehicle control. The total cell lysate was collected, and the protein levels of Aurora kinase A (AurA), p53 and 16E7 were ascertained via Western blotting. β-actin was included as a loading control.

To further assess the positive correlation between AurA and E6, we treated C33A and CaSki cells with two potent and specific AurA inhibitors (MK5108 and MLN 8237 or Alisertib) and pan-Aurora kinase inhibitors (VX 680 or Tozasertib). The cells were treated with low or high concentrations of the inhibitors: 1 nM or 10 nM MK5108, 1.2 nM or 12 nM Alisertib, as well as 1.8 nM or 18 nM Tozasertib for 24 h. The total cell lysate was collected and analyzed via Western blotting. The protein levels of AurA and p53 proteins were assessed. Our results showed that treatment of these inhibitors at high concentrations was able to reduce the expression levels of AurA and p53 proteins (Fig. 6A). Compared with the DMSO-treated samples, treatment with 1 nM (0.76 ± 0.05, *P* < 0.01) and 10 nM (0.53 ± 0.17, *P* < 0.05) of MK5108, 1.2 nM (0.45 ± 0.05, *P* < 0.05) and 12 nM (0.44 ± 0.18, *P* < 0.05) of Alisertib, as well as 1.8 nM (0.44 ± 0.20, *P* < 0.05) and 18 nM (0.45 ± 0.17, *P* < 0.05) of Tozasertib, induced a significant decreased expression level of AurA. Treatment with the inhibitors also induced an increased p53 expression in CaSki cells: 1 nM (6.12 ± 0.76, *P* < 0.01) and 10 nM (5.73 ± 4.64, *P* < 0.05) of MK5108, 1.2 nM (2.67 ± 0.32, *P* < 0.01) of Alisertib, as well as 18 nM (5.13 ± 0.05, *P* < 0.05) Tozasertib. In C33A cells, the protein levels of AurA and p53 were not affected by the treatment using these inhibitors.

As Alisertib showed an efficient inhibitory effect in CaSki cells, we further testify the efficacy of this inhibitor in HPV-positive (HeLa and SCC152 cells) and HPV-null SCC25 cells. The cells were treated with 1.2 nM or 12 nM Alisertib for 24 h. Our immunoblots showed a marginal decrease of AurA, p53, and 16E7 protein levels in SCC152 cells upon treatment with 12 nM Alisertib. However, the treatment of Alisertib, either at 1.2 nM or 12 nM, did not affect the AurA, p53, and 16E7 protein levels in HeLa and SCC25 cells (Fig. 6B). These results indicate that Aurora kinase inhibitors possess a differential ability in suppressing AurA and HPV oncoprotein levels in HPV-positive cells.

### Depletion of AurA decreased the ability of HPV-positive cells to invade.

**FIG 7:**
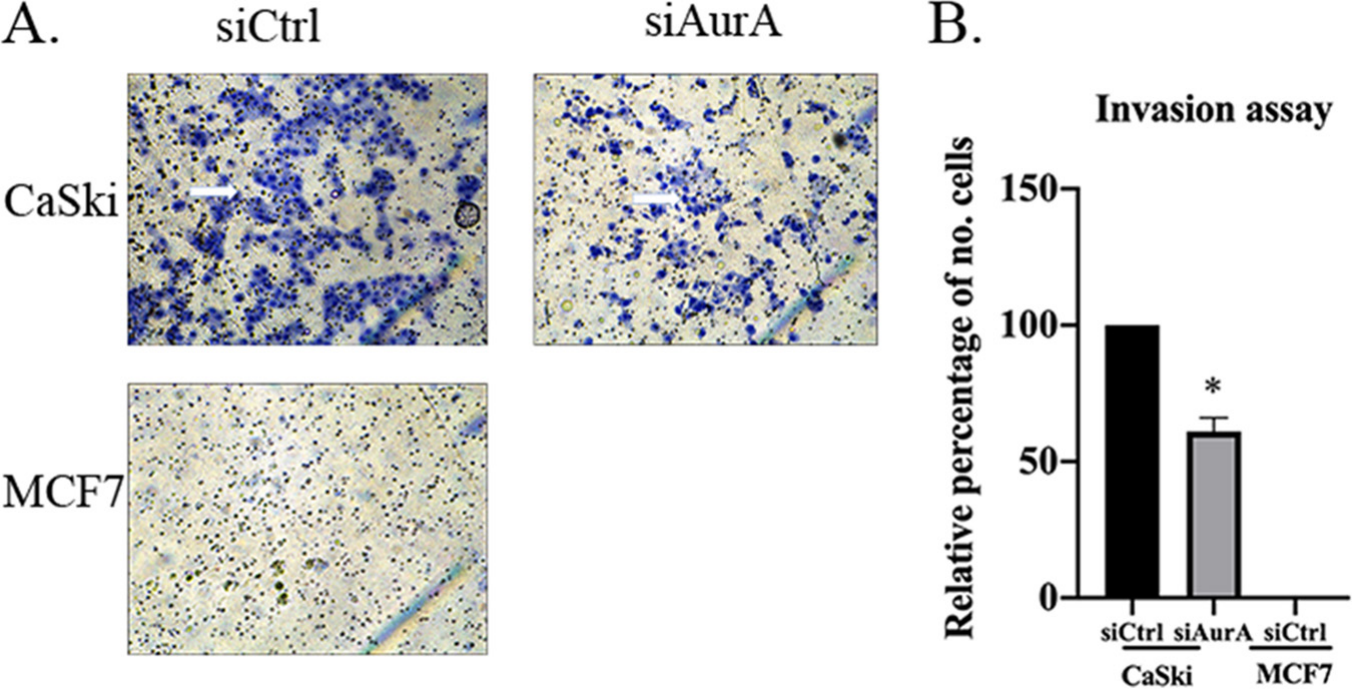
The invasion ability of HPV-positive cells with different AurA expressions. (A) The representative images of invasion capacity of the CaSki and MCF7 cells that transfected short interference RNA against AurA (siAurA) or control (siCtrl) for 72 h. The white arrows indicate the invaded cells. (B) The number of invaded CaSki and MCF7 cells was analyzed and quantitated using ImageJ. The invaded cells were observed and calculated with 10 randomly fields under ×100 magnification by Leica microscope. Note that the CaSki cells transfected with siAurA showed a significant decrease in invasion ability.

In order to further elucidate the contributing role of AurA in promoting the invasion of HPV-positive cells, we performed a Matrigel invasion assay. CaSki cells were either transfected with siRNA targeting control (siCtrl) or specifically against AurA (siAurA). After 72 h, the cells were seeded onto the Matrigel invasion chamber. As a negative control, we included noninvasive MCF7 breast cancer cells. We found that CaSki cells with AurA downregulated displayed a lower invasive ability (61.0% ± 5.0%, *P* < 0.05) compared with the siCtrl-transfected CaSki cells (Fig. 7). Altogether, these results revealed the AurA-E6 association promotes carcinogenesis, particularly enhancing the ability of HPV-positive cells to invade.

## DISCUSSION

The multifunctional E6 oncoprotein-encoded by cancer-causing human papillomavirus (HPV) can interact with numerous cellular proteins, allowing E6 to perturb normal cellular function in favor of viral genome and host cell survival. On top of the extended list of E6 target proteins, we and the other team identified AurA as a genuine interacting partner of E6, and this interaction allows the level of E6 to be enhanced by AurA (16). However, the mode and consequence of the interaction have yet to be clearly elucidated previously. We carefully characterized the amino acid residues important for AurA-E6 complex formation and elucidated the function of AurA-E6 association in promoting HPV-mediated carcinogenesis.

We identified that AurA as a potential interacting partner of E6 through an *in silico* approach. Our findings from a series of *in vitro* binding assays agreed with the computer-aided prediction. Even though E7-encoded by HPV16 also harbor AurA recognition motif, the binding affinity between E7 and AurA was much weaker compared to E6. As prediction software showed that E6-encoded by HPV11 (HPV11E6) did not harbor AurA recognition motif, which was also evident by our *in vitro* binding assay. We initially thought that the lack of PDZ binding motif of E6 (E6-PBM) in HPV11E6 could contribute to the loss of AurA binding. However, our binding assays showed the integrity of E6-PBM did not affect AurA binding to E6. Rather, our study recognized previously undefined region of E6, immediately upstream of E6-PBM, at amino acid residues T140 to S145, is important for the AurA-E6 complex formation. This demonstrates the conserved association between AurA and E6-encoded by the cancer-causing HPV genotypes is important for HPV to exert its oncogenicity.

The previous finding showed that HPV16E6 enhances the expression of AurA (16). Even though we did not see such an increment, the downregulation of HPV oncoproteins in HPV-containing cells led to a decreased AurA protein expression. Moreover, AurA overexpression can enhance the expression of HPV16E6 protein in a dose-dependent manner, reflecting the positive correlation between AurA and E6 interaction. The aberrant expression of AurA in cancers resulted it to play an oncogenic role in cancers. In HPV-positive cancer cells, the oncogenic association between AurA and E6 could be important for HPV to promote carcinogenesis, perhaps from precancerous to cancerous stages. This occurs partly through maintaining the stability of E6. Previous studies showed that Alisertib, a potent AurA inhibitor, inhibits proliferation and induces apoptosis of HPV-positive cancer cells (18, 20). In concert with this, our findings showed that suppressing the expression of AurA in HPV16-positive cervical cancer cells, either using potent AurA inhibitors, like MK5108 and Alisertib, or via siRNA targeting AurA specifically, can efficiently suppress the oncogenic potential, particularly the invasive ability of the HPV-positive cells. Nonetheless, the efficacy of AurA inhibition differs in different HPV-positive cancer cells originating from different tissue tropisms. Therefore, the use of Aurora kinase inhibitors to treat HPV-positive cancers should be carefully evaluated.

We provided important molecular insight on how E6 deploy the oncogenic function of AurA. Using overexpression and downregulation through siRNA specifically against AurA approaches, we showed that AurA-E6 promoted hallmarks of cancer, including dysregulated cell cycle checkpoints, particularly at G1/S and mitotic phases of the cell cycle, as revealed by the expression of cyclin E and phosphor/acetylated-histone H3, respectively. Through these two approaches, both cyclin E and phosphor-histone H3 expressions were elevated. Our findings reflect that status of p53 expression, particularly in cancer cells with wild-type p53 or mutants p53 expressed, could influence the alteration of cellular events. In general, HPV-positive cells often retain wild-type p53 expression, including CaSki cells. While the HPV-null cervical cancer cells, including C33A cells, often harbor multiple mutations within TP53. The expression of wild-type p53 is important to regulate cell cycle checkpoints, essentially keeping mitotic duration and frequency of mitotic aberrancies checked (21). In CaSki cells, the depletion of AurA and E6 resulted in a markedly elevated expression of cyclin E and phosphor/acetylated-histone H3. Previous studies showed that cyclin E interacts directly with E7-encoded by HPV16 (22) and that E7 can disrupt G1/S transition by regulating cyclin E expression at transcription and posttranscriptional levels (10). Knowing the weak association between E7 and AurA, the depletion of AurA in CaSki cells may not affect E7 expression level. Therefore, this may be insufficient to affect E7-mediated cyclin E overexpression, in which overexpression of cyclin E is a common phenomenon observed in cancers (23).

Phosphorylation of Histone H3 at residue serine 10 (S10) occurs following acetylation at residue lysine 9 (K9). The phosphorylation of Histone H3-S10 marks the initiation of chromosome condensation during cell division, and dephosphorylation of S10 occurs at the end of mitosis (24). Knockdown of AurA by RNA interference does not affect phosphorylation of Histone H3 (25), and the chromatin condensation is also one of the first cellular events that occur during apoptosis (25). The depletion of AurA and E6, and the “rescued” wild-type p53, could activate apoptosis in CaSki cells, leading to apoptotic-induced phosphorylation of H3-S10 and chromatin condensation (26). On top of this, the elevated cyclin E expression may instigate replication stress and genome instability (21), which then subsequently activate apoptosis and DNA damage pathways, halting cancer progression and invasion.

Meanwhile, our results showed that, in C33A cells, the co-expression of AurA and E6 does not disrupt the cyclin E expression. Nonetheless, AurA and E6 synergistically induces an increased phosphor-histone H3-S10 expression. The TP53 mutations in C33A cells, which lead to “gain-of-functions” of p53 protein may also instigate upregulation of cyclin E and mitotic aberrancies. This may further exacerbate replication stress and accumulation of mitotic errors (21). In addition, as p53 also play a role as a transcription regulator, this “gain-of-function” of p53 allows the upregulation of transcription of its target genes, including the monocytic leukemia Zinc Finger (*MOZ*), encodes for a histone acetyltransferase, which is responsible to activate histone H3. The expression of *MOZ* gene, in turn, increases gene transcription (27). Therefore, the upregulated and activated Histone H3 in C33A cells may not be due to overexpression of AurA and E6 directly. It could be enhanced by E6 and/or AurA, on top of the expression of p53 mutants. To recapitulate the role of AurA and E6 in regulating the expression of Histone H3, primary keratinocytes or normal immortalized cell lines that carry wild-type p53 can be used in the future.

In conclusion, our findings revealed that through direct interaction between AurA and E6 in the nucleus, the cell cycle checkpoints of the HPV-positive cells were perturbed. Our data suggest an important implication that disruption of the association between AurA and E6 pose a therapeutic value. Even though the inhibitors have marched to clinical trials, these therapeutics are often used for patients with hematologic and solid tumors. Their efficacy in treating patients diagnosed with HPV-associated cancers is unclear and should be conducted in the future. A potent and specific inhibitor that can disrupt the oncogenic association between AurA and E6 could offer an effective treatment approach for HPV-positive cancer cells arise from different tissue tropism.

## MATERIALS AND METHODS

### Plasmids.

HPV-E6 expression constructs, namely, pGW1:HA-HPV-16 and -18 E6, pGEX2T:HPV-11, -16, -18, -58 E6, and 16E6 ΔPBM (encoding GST-HPV11, 16, 18, 58 E6, and 16E6 ΔPBM conjugated proteins, respectively) were generously gifted by Dr. Lawrence Banks, ICGEB. Using online Scansite freeware (19), we predicted four potential AurA recognition sites within 16 E6: S89, T140, S145, and S150. These amino acids were mutated to Alanine (A), as depicted schematically in Fig. 1A. HPV16E6 mutants (16E6 T140A, 16E6 S145A, 16E6 S150A and 16E6 S89A) were generated via GeneArt site-directed mutagenesis kit according to the manufacturer’s protocol (Invitrogen, USA). The primer pairs used to generate these expression constructs were listed in Table 1. The resulting plasmids were all verified by Sanger sequencing and transformed into E. coli strain DH5-α. These expression constructs were used in *in vitro* binding, co-immunoprecipitation, and transient expression assays. The pcDNA3.1: Flag-AurA was generated by subcloning PCR-amplified AurA into the compatible *Bam*R I and EcoR I restriction site of pcDNA3.1: Flag vector. The pcDNA3.1: HA-AurA were generously provided by Prof. Yi-ren Hong, Faculty of Medicine, Kaoshiung Medical University, Taiwan. For subcellular localization and immunoprecipitation assays, pGW1:HA-HPVE6s and pcDNA:Flag-AurA mammalian expression constructs were used.

**TABLE 1:** The sequences primers used to construct HPV16E6 mutants^*a*^

Mutation site within HPV16E6	Primer sequence
S89A	
Forward	5’GAGTATAGACATTATTGTTAT**GCA**TTGTATGGAACAAC-3′
Reverse	5′-ATGTTGTTCCATACAA**TGC**ATAACAATAATGTCTATAC-3′
T140A	
Forward	5′-AATATAAGGGGTCGGTGG**GCA**GGTCGATGTATGTCT-3′
Reverse	5′-CAAGACATACATCGACC**TGC**CCACCGACCCCTTATA-3′
S145A	
Forward	5′-GGACCGGTCGATGTATG**GCA**TGTTGCAGATCATCAAG-3′
Reverse	5′-CTTGATGATCTGCAACA**TGC**CATACATCGACCGGTCC-3′
S150A	
Forward	5′-GTCTTGTTGCAGATCA**GCA**AGAACACGTAGAGAAAC-3′
Reverse	5′-GTTTCTCTACGTGTTCT**TGC**TGATCTGCAACAAGAC-3′

a The intended mutation sites are in bold and underlined.

### Cell lines.

HeLa (HPV18 cervical cancer cells), CaSki (HPV16 cervical cancer cells), human embryonic kidney (HEK) 293 (HPV-null epithelial cells), C33A (HPV-null cervical cancer cells), U-2 OS (HPV-null human osteosarcorma cells), HeKa (HPV-null primary keratinocytes), SCC25 (HPV-null human head and neck head and neck squamous cell carcinoma [HNSCC] cell line), and SCC152 (HPV16-positive human HNSCC cell line) were purchased from the American Type Culture Collection (ATCC) and maintained in Dulbecco's modified Eagle medium (DMEM) supplemented with 10% fetal bovine serum (FBS, GIBCO) at 37°C in a humidified incubator with 5% CO_2_. Identities of these human cell lines were validated by short tandem repeat (STR) profiling using the AmpFlSTR Identifiler Plus PCR Amplification Kit (Thermo Fisher Scientific) with the Applied Biosystems 3500 Series Genetic Analyzer, and analyzed by GeneMapper Software 5 (Applied Biosystems). The STR profile of all cells lines showed >88% concordance with their reference profiles in the ATCC cell line database.

### Fusion protein purification and *in vitro* binding assays.

The purification of GST-tagged fusion proteins and *in vitro* translation of AurA were performed as described previously (28). Transformants harboring pGEXT2T:HPVE6s constructs were grown overnight. The culture was then inoculated into fresh medium and incubated for 1 h. Recombinant protein expression was then induced by addition of isopropyl-β-d-thiogalactopyranoside (IPTG, Sigma) to a final concentration of 1 mM and incubated for a further 3 h. The bacteria were harvested and lysed with cold 1× PBS containing 1% Triton X-100, and sonicated twice for 30 s. The supernatants were collected and incubated with glutathione-conjugated agarose resin on a rotating wheel overnight at 4°C. After extensive washing, the amount of immobilized GST fusion proteins were analyzed by sodium dodecyl sulfate-polyacrylamide gel electrophoresis (SDS-PAGE) and stained by GelCode Blue Stain Reagent (Thermo Fisher Scientific).

AurA protein was translated *in vitro* using the TNT Coupled Reticulocyte Lysate System (Promega), according to the manufacturer’s recommendation. Equal amounts of *in vitro*-translated AurA were added to purified GST fusion proteins and incubated for 1 h at room temperature. After being washed 3 times with PBS containing 0.1% Tween 20, the bound proteins were subjected to SDS-PAGE and Western blotting for detection of AurA.

### Immunofluorescence assay.

This assay is carried out as described previously (29). Approximately 2 × 10^5^ U-2 OS cells were plated onto coverslips. The cells were transfected with HA-tagged 16 E6 and Flag-tagged AurA. Cells were fixed with ice-cold absolute methanol 24 h after transfection and incubated with primary antibody against HA (Roche), Flag (Santa Cruz Biotechnology), and AurA (Cell Signaling), followed by Alexa Fluor 568-conjugated anti-rabbit secondary antibody and Alexa Fluor 488-conjugated anti-mouse secondary antibodies (Thermo Fisher Scientific), and counterstained in 4′,6-diamidino-2-phenylindole (DAPI). The subcellular location of HPV16 E6 and AurA were examined under a fluorescence microscope (Nikon).

### ADP-Glo AurA kinase assay.

The kinase reaction was carried out by incubating approximately 2 μg of purified AurA and with equal amounts of purified HPVE6s GST fusion proteins in the presence 30 μL of kinase reaction buffer containing 25 mM Tris (pH 7.5), 10 mM MgCl_2_, 3.5 mM NaCl, and 75 μM ATP for 1 h at 25°C. The AurA kinase activity was measured using ADP-Glo Kinase Assay (Promega), according to the manufacturer’s recommendations. Briefly, the kinase reaction was mixed with ADP-Glo Reagent at a ratio of 1:1 and incubated in a white base 384-well plate for 1 h. Following this, one volume of kinase detection reagent was added and incubated for an additional 1 h at 25°C in dark. The amount of luminescence ADP produced was measured using VICTOR Multilabel Plate Reader (PerkinElmer).

### Transient transfection and transfection of specific siRNA against AurA.

C33A and U-2 OS cells were transfected using Lipofectamine 3000 transfection reagent (Thermo Fisher Scientific) according to the manufacturer’s protocol. Briefly, approximately 3 × 10^5^ cells were seeded into a 6-well plate. On a subsequent day, approximately 2 μg of DNA, P3000 and Lipofectamine 3000 were diluted in DMEM reduced serum media (Thermo Fisher Scientific). The mixture was then added to cells and incubated for 5 h at 37°C. The medium was then replenished with fresh DMEM media containing 10% FBS and incubated overnight.

HEK 293 cells were transfected using the calcium phosphate precipitation method (30). The cells (3 × 10^5^ cells) were seeded into a 6-well plate and incubated for 24 h. The calcium phosphate (Ca_3_(PO4)_2_)-DNA complex was prepared by adding 3 μg of DNA, and 11 μL calcium chloride (250 mM) was diluted in 97.5 μL 1 mM Tris (pH 7.9)-0.1 mM EDTA (TE) buffer in one tube and then mixed. The mixture of DNA was then added to 100 μL 2 × HEPES-Buffered Saline (HBS) (280 mM NaCl, 50 mM HEPES, and 1.5 mM sodium phosphate) pH 7.1 to 7.2 drop by drop and incubated for 30 min at room temperature. The mixture was then added to cells and incubated overnight.

HeLa, CaSki, C33A cells were transfected with specific siRNA targeting AurA/AIK (Cell Signaling Technology, USA) using Lipofectamine LTX transfection reagent (Thermo Fisher Scientific, USA), as described previously (29). To prepare the siRNA-liposome complex, 133 nM siAurA (Cell Signaling Technology, USA) and Lipofectamine LTX reagent were diluted in serum-free Opti-MEM reduced serum media. The mixture was then added to cells and incubated for 5 h at 37°C. The medium was then replenished with fresh Opti-MEM media containing 10% FBS and incubated for 72 h.

### Western blotting.

The total cell lysate was collected by lysing cell pellets with 2× sodium dodecyl sulfate (SDS) sample buffer and boiled at 95°C for 5 min. Total protein lysate was resolved by SDS-PAGE and transferred onto a polyvinylidene fluoride (PVDF) membrane (GE Healthcare). The membrane was then probed with specific primary antibodies overnight at 4°C: phosphor-AurA (T288), phosphor-AKT, AKT, AurA and HA (Cell Signaling Technology); MMP2 (Abcam); β-galactosidase (Promega); p53 (DO-1), phosphor-acetylate-Histone H3, Histone H3, phosphor-ERK1/2, ERK1/2, cyclin E, cyclin B, Vimentin, Flag, and β-actin (Santa Cruz Biotechnology). Subsequently, the immunoblots were incubated with the appropriate HRP-conjugated secondary antibodies. Blots were visualized using Clarity Western ECL Substrate (Bio-Rad) and images were captured using a ChemiDoc Imaging System (Bio-Rad). Beta (β)-actin served as loading controls for the blots. Band intensities were quantified by Image Lab Software (Bio-Rad) and normalized using the corresponding loading controls.

### Cell invasion assay.

The cell invasion assay was performed as described previously (31). BioCoat Matrigel Invasion Chamber (Corning, USA) was used to detect the invasive capacity of HPV-positive cells (CaSki) upon AurA knockdown. MCF7, a noninvasive breast cancer cell line was included as a negative control. Approximately 3 × 10^5^ of the cells were seeded into a 6-well plate and incubated for 24 h. These cells were then transfected with specific siRNA against AurA (siAurA) as described above. CaSki and MCF7 cells were then trypsinized and resuspended in serum-free DMEM media. Each CaSki and MCF7 cell (5 × 10^4^ cells) was seeded into the upper chamber containing serum-free DMEM. The DMEM medium containing 20% FBS was added to the lower chamber of the invasion chamber. After 24 h of incubation, the upper chamber was washed with PBS and wiped using autoclaved cotton swabs to remove noninvaded cells. The cells on the sides of the membrane in the lower chamber were fixed using 500 μL absolute methanol, and two drops of methanol were added into the upper section for 5 min. The fixed cells were stained with hematoxylin for 45 min and washed with double-distilled water (ddH_2_O), followed by washing with 80% ethanol. The chamber was inverted and allowed to air dry overnight. The stained membrane was excised, immersed in xylene and mounted on the glass slide with ProLong Diamond Antifade Mountant (Invitrogen, USA). The slides were examined at ×100 magnification using a light microscope (Leica, Germany). A random 10 fields of images were taken and the number of invaded cells was calculated using ImageJ software.

### Data availability.

The data and materials are available upon request.

## References

[1] Werness BA, Levine AJ, Howley PM. 1990. Association of human papillomavirus types 16 and 18 E6 proteins with p53. Science 248:76–79. 10.1126/science.2157286. 2157286

[2] Ganti K, Broniarczyk J, Manoubi W, Massimi P, Mittal S, Pim D, Szalmas A, Thatte J, Thomas M, Tomaić V, Banks L. 2015. The human papillomavirus E6 PDZ binding motif: from life cycle to malignancy. Viruses 7:3530–3551. 10.3390/v7072785. 26147797 PMC4517114

[3] Gonzalez SL, Stremlau M, He X, Basile JR, Münger K. 2001. Degradation of the retinoblastoma tumor suppressor by the human papillomavirus type 16 E7 oncoprotein is important for functional inactivation and is separable from proteasomal degradation of E7. J Virol 75:7583–7591. 10.1128/JVI.75.16.7583-7591.2001. 11462030 PMC114993

[4] Menges CW, Baglia LA, Lapoint R, McCance DJ. 2006. Human papillomavirus type 16 E7 up-regulates AKT activity through the retinoblastoma protein. Cancer Res 66:5555–5559. 10.1158/0008-5472.CAN-06-0499. 16740689 PMC9048434

[5] Pim D, Massimi P, Dilworth SM, Banks L. 2005. Activation of the protein kinase B pathway by the HPV-16 E7 oncoprotein occurs through a mechanism involving interaction with PP2A. Oncogene 24:7830–7838. 10.1038/sj.onc.1208935. 16044149

[6] Spangle JM, Münger K. 2010. The human papillomavirus type 16 E6 oncoprotein activates mTORC1 signaling and increases protein synthesis. J Virol 84:9398–9407. 10.1128/JVI.00974-10. 20631133 PMC2937655

[7] Dyson N, Guida P, Münger K, Harlow E. 1992. Homologous sequences in adenovirus E1A and human papillomavirus E7 proteins mediate interaction with the same set of cellular proteins. J Virol 66:6893–6902. 10.1128/JVI.66.12.6893-6902.1992. 1331501 PMC240306

[8] Watanabe S, Sato H, Komiyama N, Kanda T, Yoshiike K. 1992. The E7 functions of human papillomaviruses in rat 3Y1 cells. Virology 187:107–114. 10.1016/0042-6822(92)90299-5. 1310552

[9] Spanos WC, Geiger J, Anderson ME, Harris GF, Bossler AD, Smith RB, Klingelhutz AJ, Lee JH. 2008. Deletion of the PDZ motif of HPV16 E6 preventing immortalization and anchorage-independent growth in human tonsil epithelial cells. Head Neck 30:139–147. 10.1002/hed.20673. 17657785 PMC2600880

[10] Martin LG, Demers GW, Galloway DA. 1998. Disruption of the G1/S transition in human papillomavirus type 16 E7-expressing human cells is associated with altered regulation of cyclin E. J Virol 72:975–985. 10.1128/JVI.72.2.975-985.1998. 9444990 PMC124568

[11] Vliet-Gregg PA, Hamilton JR, Katzenellenbogen RA. 2013. NFX1-123 and human papillomavirus 16E6 increase Notch expression in keratinocytes. J Virol 87:13741–13750. 10.1128/JVI.02582-13. 24109236 PMC3838236

[12] Nikonova AS, Astsaturov I, Serebriiskii IG, Dunbrack RL, Golemis EA. 2013. Aurora A kinase (AURKA) in normal and pathological cell division. Cell Mol Life Sci 70:661–687. 10.1007/s00018-012-1073-7. 22864622 PMC3607959

[13] Tang A, Gao K, Chu L, Zhang R, Yang J, Zheng J. 2017. Aurora kinases: novel therapy targets in cancers. Oncotarget 8:23937–23954. 10.18632/oncotarget.14893. 28147341 PMC5410356

[14] D'Assoro AB, Liu T, Quatraro C, Amato A, Opyrchal M, Leontovich A, Ikeda Y, Ohmine S, Lingle W, Suman V, Ecsedy J, Iankov I, Di Leonardo A, Ayers-Inglers J, Degnim A, Billadeau D, McCubrey J, Ingle J, Salisbury JL, Galanis E. 2014. The mitotic kinase Aurora-A promotes distant metastases by inducing epithelial-to-mesenchymal transition in ERα+ breast cancer cells. Oncogene 33:599–610. 10.1038/onc.2012.628. 23334326 PMC4058768

[15] Xia Z, Wei P, Zhang H, Ding Z, Yang L, Huang Z, Zhang N. 2013. AURKA governs self-renewal capacity in glioma-initiating cells via stabilization/activation of β-catenin/Wnt Signaling. Mol Cancer Res 11:1101–1111. 10.1158/1541-7786.MCR-13-0044. 23761169

[16] Guo Y, Ma J, Zheng Y, Li L, Gui X, Wang Q, Meng X, Shang H. 2016. HPV16 E6 upregulates Aurora A expression. Oncol Lett 12:1387–1393. 10.3892/ol.2016.4786. 27446442 PMC4950527

[17] Gabrielli B, Bokhari F, Ranall MV, Oo ZY, Stevenson AJ, Wang W, Murrell M, Shaikh M, Fallaha S, Clarke D, Kelly M, Sedelies K, Christensen M, McKee S, Leggatt G, Leo P, Skalamera D, Soyer HP, Gonda TJ, McMillan NAJ. 2015. Aurora A is critical for survival in HPV-transformed cervical cancer. Mol Cancer Ther 14:2753–2761. 10.1158/1535-7163.MCT-15-0506. 26516156

[18] Shaikh MH, Idris A, Johnson NW, Fallaha S, Clarke DTW, Martin D, Morgan IM, Gabrielli B, McMillan NAJ. 2018. Aurora kinases are a novel therapeutic target for HPV-positive head and neck cancers. Oral Oncol 86:105–112. 10.1016/j.oraloncology.2018.09.006. 30409290

[19] Ehrenberger T, Cantley LC, Yaffe MB. 2015. Computational prediction of protein-protein interactions. Methods Mol Biol 1278:57–75. 10.1007/978-1-4939-2425-7_4. 25859943 PMC4435844

[20] Yumol J, Gabrielli B, Tayyar Y, McMillan NA, Idris A. 2020. Smart drug combinations for cervical cancer: dual targeting of Bcl-2 family of proteins and aurora kinases. Am J Cancer Res 10:3406–3414. 33163279 PMC7642645

[21] Kok YP, Guerrero Llobet S, Schoonen PM, Everts M, Bhattacharya A, Fehrmann RSN, van den Tempel N, van Vugt MATM. 2020. Overexpression of Cyclin E1 or Cdc25A leads to replication stress, mitotic aberrancies, and increased sensitivity to replication checkpoint inhibitors. Oncog 9:1–15. 10.1038/s41389-020-00270-2 PMC7542455 33028815

[22] Nguyen CL, Münger K. 2008. Direct association of the HPV16 E7 oncoprotein with cyclin A/CDK2 and cyclin E/CDK2 complexes. Virology 380:21–25. 10.1016/j.virol.2008.07.017. 18718623 PMC2574417

[23] Erlandsson F, Wählby C, Ekholm-Reed S, Hellström AC, Bengtsson E, Zetterberg A. 2003. Abnormal expression pattern of cyclin E in tumour cells. Int J Cancer 104:369–375. 10.1002/ijc.10949. 12569561

[24] Van Hooser A, Goodrich DW, David Allis C, Brinkley BR, Mancini MA. 1998. Histone H3 phosphorylation is required for the initiation, but not maintenance, of mammalian chromosome condensation. J Cell Sci 111:3497–3506. 10.1242/jcs.111.23.3497. 9811564

[25] Giet R, Glover DM. 2001. Drosophila Aurora B kinase is required for histone H3 phosphorylation and condensin recruitment during chromosome condensation and to organize the central spindle during cytokinesis. J Cell Biol 152:669–682. 10.1083/jcb.152.4.669. 11266459 PMC2195771

[26] Park CH, Kim KT. 2012. Apoptotic phosphorylation of Histone H3 on Ser-10 by protein kinase Cδ. PLoS One 7. 10.1371/journal.pone.0044307. PMC3440438 22984491

[27] Prives C, Lowe SW. 2015. Mutant p53 and chromatin regulation. Nature 525:199–200. 10.1038/nature15212. 26331537 PMC4683398

[28] Boon SS, Tomaić V, Thomas M, Roberts S, Banks L. 2015. Cancer-causing human papillomavirus E6 proteins display major differences in the phospho-regulation of their PDZ interactions. J Virol 89:1579–1586. 10.1128/JVI.01961-14. 25410862 PMC4300763

[29] Boon SS, Chen Z, Li J, Lee KYC, Cai L, Zhong R, Chan PKS. 2019. Human papillomavirus type 18 oncoproteins exert their oncogenicity in esophageal and tongue squamous cell carcinoma cell lines distinctly. BMC Cancer 19:1211. 10.1186/s12885-019-6413-7. 31830929 PMC6909509

[30] Wigler M, Pellicer A, Silverstein S, Axel R, Urlaub G, Chasin L. 1979. DNA-mediated transfer of the adenine phosphoribosyltransferase locus into mammalian cells. Proc Natl Acad Sci USA 76:1373–1376. 10.1073/pnas.76.3.1373. 286319 PMC383253

[31] Boon SS, Xia C, Lim JY, Chen Z, Law PTY, Yeung ACM, Thomas M, Banks L, Chan PKS. 2020. Human papillomavirus 58 E7 T20I/G63S variant isolated from an East Asian population possesses high oncogenicity. J Virol 94. 10.1128/JVI.00090-20. PMC7108839 31996427

